# Aging alone or combined with obesity increases white adipose tissue inflammatory status in male mice

**DOI:** 10.1038/s41598-024-67179-3

**Published:** 2024-07-15

**Authors:** Lorrine Bournot, Thomas Payet, Flavie Sicard, Thomas Breniere, Julien Astier, Julien Roux, Bruno Bariohay, Jean-François Landrier

**Affiliations:** 1 Aix-Marseille Université, C2VN, INRAE, INSERM, 13000 Marseille, France; 2 Biomeostasis, 13070 La Penne Sur Huveaune, France; 3 PhenoMARS, CriBiom, Marseille, France; 4 C2VN, UMR 1260 INRAE/1263 INSERM/Aix Marseille Université, 27 Bd Jean Moulin, 13385 Marseille Cedex 05, France

**Keywords:** Inflammatory, Vitamin D, Aging, Obesity, Mice, Adipose tissue, Physiology, Endocrinology

## Abstract

White adipose tissue (WAT) has been recognized as a fundamental and crucial organ of interest in research focusing on inflammation during obesity or aging. WAT is also proposed as a significant component of cholecalciferol and 25-hydroxyvitamin D (25(OH)D) storage, which participates in the decrease of 25(OH)D plasma levels reported during aging and obesity. In the present study, we evaluated WAT and plasma cholecalciferol and 25(OH)D content together with inflammatory status to highlight the putative relationship between vitamin D status and inflammatory process during aging alone or combined with obesity. Circulating cholecalciferol and 25(OH)D and the stored quantity of cholecalciferol and 25(OH)D in WAT were quantified in young and old mice fed a control or obesogenic diet. The inflammation was assessed by measuring plasma inflammatory cytokines, mRNA, and microRNAs inflammatory-associated in WAT. The combination of aging and obesity decreased 25(OH)D plasma levels but did not modify circulating inflammatory markers. A cumulative effect of aging and obesity was observed in WAT, with rising mRNA inflammatory cytokines, notably *Ccl5* and *Tnf*. Interestingly, aging and obesity-associated were also characterized by increased inflammatory microRNA expression. The inflammatory parameters in WAT were negatively correlated with the plasma 25(OH)D but positively correlated with the quantity of cholecalciferol and 25(OH)D in WAT. These results support the cumulative effect of obesity and aging in aggravation of WAT inflammation and suggest that accumulation of cholecalciferol and 25(OH)D in WAT could constitute a mechanism to counteract WAT inflammation during aging and obesity.

## Introduction

Aging and obesity are significant challenges to global public health as they contribute to the development of metabolic diseases that are closely related to chronic low-grade inflammation^1^. In recent years, significant research has explored the intricate interplay between aging, obesity, inflammation, and vitamin D levels. Examining these interrelated processes is paramount and crucial for developing a comprehensive approach to understanding metabolic dysregulation and improving overall health outcomes associated with aging.

White adipose tissue (WAT) is a complex and dynamic organ crucial to health, metabolism, and longevity^2^. This tissue can produce and secrete different hormones and cytokines that regulate and participate in inflammation. It is well established that WAT drives the chronic inflammatory response observed in overweight and obesity. Indeed, fat mass accretion leads to low-grade increased cytokine production (interleukin (IL) 6, Tumoral necrosis factor (TNF), etc.) and chemokines (monocyte chemoattractant protein 1 (MCP1), regulated on activation, normal T cell expressed and secreted (Rantes), etc.)^3^. Such metabolic inflammation is also characterized by increased production of microRNAs (miRNAs)^4–6^. MiRNAs are small non-coding RNA with a functional regulatory role in several biological processes^7^. Indeed, they selectively target numerous messenger RNA (mRNA) molecules to repress their expression, thus modulating a wide array of signaling pathways, which encompasses the regulation of obesity but also aging-related processes^8,9^. Aging is characterized by a progressive decline in physiological functions and an altered metabolic profile with a shift in fat distribution from subcutaneous to visceral fat depots, contributing to age-associated diseases' development^10^, including inflammatory processes. Moreover, overweight and inflammatory mediators accelerate the progression of age-associated diseases, underscoring the significant impact of adipose tissue in aging^2,11^.

Vitamin D, traditionally known for its role in calcium homeostasis and bone health, has emerged as a potential modulator of adipose tissue function^12–14^. Indeed, adipocytes express enzymes of vitamin D metabolism, store vitamin D in their lipid droplets, and metabolize vitamin D, which can mediate anti-inflammatory effects in adipose tissue^12,13,15–17^. Our team has notably shown that vitamin D limited cytokine, chemokine, and miRNA expression in adipocytes or WAT in humans and mice^18–24^. Several studies have shown that vitamin D deficiency, defined by 25-hydroxyvitamin D (25(OH)D) plasma levels below 50 nmol/L^25^, was negatively correlated with inflammatory markers^26^. Additionally, it is well established that aging is associated with low circulating levels of 25(OH)D^27,28^.

Thus, given the coexistence of low levels of 25(OH)D observed during obesity or aging, and based on the anti-inflammatory effect of vitamin D, notably at the adipose level, we aimed at evaluating the combined impact of aging and obesity on inflammation status and the correlation with cholecalciferol and 25(OH)D levels in plasma and WAT. To this aim, we compared young and aged mice fed with a control diet (CD) or high-fat diet (HF).

## Material and methods

### Ethics statement and animal experiments

All experimental procedures were approved by the Committee of Aix-Marseille University and the French Ministry of Research (APAFIS#28753-202012211103783). All methods were performed according to the relevant guidelines and regulations. The study is reported by ARRIVE guidelines (https://arriveguidelines.org). Male C57BL/6JRJ mice, aged two months (n = 20) or eighteen months (n = 20), were obtained from Janvier Labs (Le Genest-Saint-Isle, France) and fed ad libitum with control diet (CD: AIN-93G, Augy, France) or high-fat diet containing 45% of energy from lipids (HF: 251HF, Augy, France) with unrestricted access to drinking water, for six months. Thus, at the end of the protocol, mice were respectively aged eight months (young mice) and twenty-four months (old mice) fed either CD or HF diet. Diets contained 1250 IU/kg of cholecalciferol for the CD diet and 1500 IU/kg for the HF diet. Mice ingested 4 IU of cholecalciferol during the study period, corresponding to mice's estimated nutrient requirements^29^. All the groups were maintained at 22 °C under a 12-h light, 12-h dark cycle, and a 20% humidity level. Twice a week, mice's body weight and food intake were measured. A whole-body composition, including fat mass (Table 1), was measured at baseline and two days before the end of the protocol using quantitative magnetic resonance in awake animals (Minispec Analyzer LF50, Bruker, Germany). At the end of the study, mice had a food restriction overnight, and blood was collected by cardiac puncture with isoflurane anesthesia; plasma was isolated by centrifugation at 3000 rcf for 15 min at four °C and was stored at − 80°C. Mice were euthanized by cervical dislocation, and peri-epididymal white adipose tissue was collected, weighted (mentioned as WAT mass in Table 1), and stored at − 80 °C.

**Table 1:** Morphological parameters of mice.

Parameters	Mean ± SEM				2way ANOVA
	Young CD	Young HF	Old CD	Old HF	
Body weight (g)	29.63^a^ ± 1.1	35.56^b^ ± 3.29	32.77^a,b^ ± 3.78	40.24^c^ ± 6.13	A/D
WAT mass (g)	0.78^a^ ± 0.11	1.36^b^ ± 0.65	0.79^a^ ± 0.43	1.89^c^ ± 0.60	D
Fat mass (g)	4.38^a^ ± 0.76	8.56^b^ ± 2.75	4.46^a^ ± 2.02	10.93^b^ ± 4.10	D
D3 plasma (ng/ml)	7.21^a^ ± 3.2	6.18^b^ ± 3.53	6.68^a^ ± 5.24	7.61^a^ ± 5.22	
25(OH)D3 plasma (ng/ml)	25.11^a^ ± 4.52	19.41^b^ ± 4.27	17.98^b^ ± 4.01	19.14^b^ ± 3.99	A/A.D
D3 quantity in WAT (ng)	3.34^a^ ± 1.2	3.29^b^ ± 1.40	7.31^a,b^ ± 4.04	9.86^b^ ± 4.90	A
25(OH)D3 quantity in WAT (ng)	0.63^a^ ± 0.18	1.37^b^ ± 0.27	0.96^a,b^ ± 0.31	2.12^c^ ± 2.12	A/D

At the end of the study, mice were weighed, the total fat mass (fat mass) was measured by quantitative magnetic resonance, and the peri-epididymal white adipose tissue was collected and weighted (WAT mass). Systemic concentrations of cholecalciferol and 25(OH)D and adipose quantities were analyzed by LC–MS/MS. Values are expressed as means ± SEM. Values not sharing the same letter (a, b, or c) were significantly different in Fisher’s LSD post hoc test p < 0.05 between the control group (CD) and high-fat group (HF) for young and aged mice. A, age effect in two-way ANOVA analysis (p < 0.05); D, diet effect in two-way ANOVA analysis (p < 0.05); A.D, the interaction between time and diet in two-way ANOVA analysis (p < 0.05).

### Cholecalciferol and 25(OH)D3 quantification in plasma and adipose tissue by LC–MS/MS

Quantification was performed using liquid chromatography-tandem mass spectrometry, an LC–MS/MS Hypersil Gold® C18 column, TSQ Fortis Plus™, and Chromeleon7™ software (Thermo Fisher Scientific, Waltham, USA) according to the previously described protocol^30^. Cholecalciferol and 25(OH)D was expressed as a concentration in adipose tissue or as a quantity in ng of cholecalciferol or 25(OH)D present in the total mass od epidydimal WAT, by multiplying the concentration by the mass of epidydimal WAT.

### Panel of inflammatory cytokines quantification in plasma by flow cytometry

To determine the concentration of systemic inflammatory cytokines in the plasma of mice groups, flow cytometry was used, according to the manufacturer protocol of *Mouse inflammation panel* and *Mouse T Helper Cytokine panel version 3*, to quantify IL-8, IFN-γ, TNF, and IL-1β (Bio Legend San Diego, California, USA).

### RNA extraction and real-time PCR

Total RNA from peri-epididymal white adipose tissue was extracted using TRIzol reagent (Thermo Fischer Scientific, Les Ulis, France). After that, one µg of total RNA from this tissue was utilized to synthesize cDNA using random primers and Moloney murine Leukemia virus reverse transcriptase (Thermo Fischer Scientific, Les Ulis, France). qPCR analysis used the LightCycler 480 instrument (Roche Applied Science, Penzberg, Allemagne) as previously reported^31^. qPCR reaction was conducted using a SYBR Green Master mix (PowerUp™ SYBR®, Thermo Fisher, Courtaboeuf, France). The comparative cycle threshold (CT) method was used, with 18s rRNA as the endogenous control for each group. RNA expression was always quantified in duplicate, and data were expressed using a relative ratio. The WAT miRNA expression was assessed by RT-qPCR analysis using an inflammatory panel of miRCURY LNA miRNA PCR Assays (QIAGEN, Courtabœuf, France). For each group, *Snord68* was used as the control in the comparative cycle threshold CT method, and the miRNA expression was always quantified in duplicate. Data were also expressed as a relative ratio.

### Heatmap and pathway analysis

The Heatmap analysis was assessed using the MetaboAnalyst platform^32^. The pathway analysis was performed using the Mienturnet database^33^ and the Ingenuity Pathway Analysis (IPA) platform, where we uploaded the miRNA and all their predicted targets. Pathways graphs were generated by GraphPad Prism software.

### Statistical analysis and correlation matrix

Data were expressed as the mean ± SEM. The normality of parameters has been tested using the Shapiro–Wilk test implemented in GraphPad Prism. Significant differences between all the groups were determined by a 2-ways ANOVA test with Fisher’s LSD post hoc test using GraphPad Prism. *p* < 0.05 was considered to be statistically significant. With two-way ANOVA analysis, D: diet effect (p < 0.05), A: aging effect (p < 0.05), and A.D: interaction between aging and diet (p < 0.05). To generate the correlation matrix, we used R software. All the values identified as statistically significant were in a color circle: blue for a positive correlation and red for a negative one.

## Results

### Morphological parameters and vitamin D status disturbance during aging and obesity

As previously reported^30^, young mice fed a high-fat (HF) diet exhibited a notable increase in body weight compared with young mice fed a control diet (CD) (Supplemental data 1). A similar trend was observed in aged mice subjected to HF and CD diets, thereby confirming the significant impact of the diet type, as supported by the two-way ANOVA results (D in Table 1). Additionally, the combined influence of aging and obesity was evident in this parameter, thereby substantiating an age effect (A in Table 1). The peri-epididymal white adipose tissue and overall fat mass exhibited a comparable pattern in the diet effect, but no significant age effect was observed (Table 1).

Notably, all mice in this study received an equal amount of vitamin D in their diets, as confirmed by the consistent systemic cholecalciferol levels across all experimental groups (Table 1). Interestingly, our study demonstrated a decrease in plasma 25(OH)D levels under HF diet in young and aged mice (both CD and HF-fed mice) but no combined effect of aging and obesity. However, we identified an age effect and an interaction between age and diet through the two-way ANOVA analysis (Table 1). Regarding the quantity of cholecalciferol present in the WAT, we observed an increase during aging, indicating an age effect but no effect of diet (Table 1). Conversely, a substantial increase in 25(OH)D in the WAT was noted during obesity, combined with aging and obesity. The two-way ANOVA analysis further revealed the influence of both age and diet on this particular parameter (Table 1).

### Impact of aging and obesity on systemic and WAT inflammation

**Figure 1:**
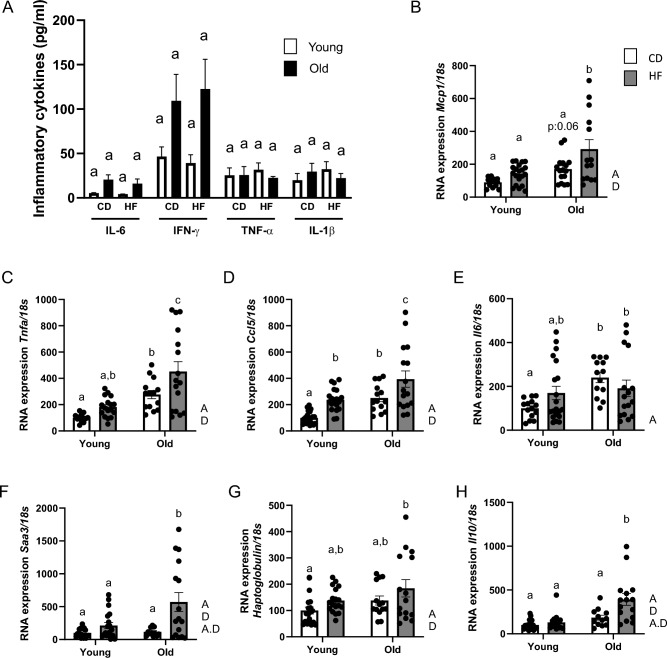
Aging and obesity impact systemic and white adipose tissue local inflammation in mice. (**A**) Systemic inflammatory cytokines measured by flow cytometry. (**B**) mRNA expression of inflammatory cytokines like Mcp1, (**C**) Tnf, (**D**) Ccl5, (**E**) Il6, (**F**) Saa3, (**G**) Haptoglobulin, and (**H**) Il10 were analyzed in WAT by qPCR and normalized by mRNA expression of 18s. Values are expressed as means ± SEM. Bars not sharing the same letter were significantly different in Fisher’s LSD post hoc test p < 0.05 between the control group (CD) and high-fat group (HF) for young and aged mice. A, Age effect in two-way ANOVA analysis (p < 0.05); D, diet effect in two-way ANOVA analysis (p < 0.05); A⋅D the interaction between time and diet in two-way ANOVA analysis (p < 0.05).

Different inflammatory cytokines were quantified in plasma. As depicted in Fig. 1A, the systemic inflammatory response remained unaltered across all experimental groups. However, within the WAT, a notable upregulation in the expression of several inflammatory cytokine mRNAs during aging and obesity was observed (Fig. 1B–H). Indeed, the mRNA expression levels of *Mcp1*, *Saa3*, *Haptoglobin*, and *Il10* exhibited a significant increase with the combined influence of aging and obesity, thus confirming the individual impacts of age and diet across all experimental groups (Fig. 1B,F–H). Notably, an interaction effect was observed for the mRNA expression of *Saa3* and *Il10* (Fig. 1F,H). Moreover, the mRNA expression of *Ccl5* displayed a significant increase in HF-fed young mice, aged mice, and particularly in the group with the combination of aging and obesity, with an apparent age and diet effect noted through the two-way ANOVA analysis (Fig. 1B). A similar trend was observed for *Tnf*, with a significant increase in the combination of aging and obesity, and a non-significant trend was observed in HF-fed young mice (Fig. 1C). As for *Il6* mRNA expression, we only observed an age effect, with a non-significant trend in HF-fed young mice (Fig. 1E).

### Inflammatory miRNAs are differentially modulated in WAT

**Figure 2:**
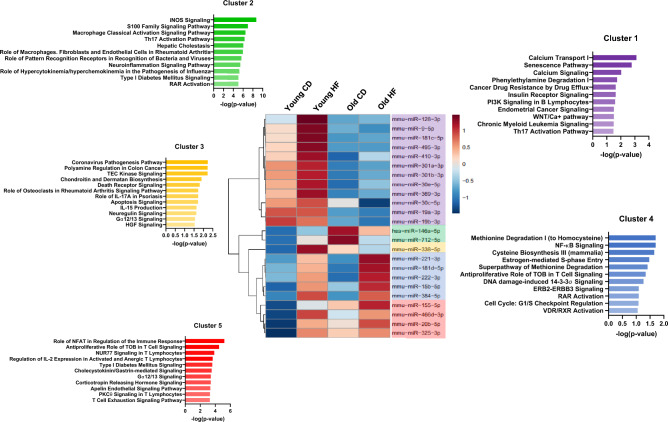
Inflammatory miRNAs are differentially modulated in WAT. A Heatmap of miRNA in WAT was generated using MetaboAnalyst, and the pathways targeted by these miRs were identified using IPA software. miRNA following the same expression pattern were clustered in five different groups. Bar charts illustrate the different pathways targeted by these clusters.

Inflammation-linked miRNA expression during aging and obesity in WAT was assessed using an inflammatory miRNA qPCR panel. This enabled us to generate a heatmap highlighting differentially expressed miRNAs (Supplemental data 2 and 3). Only miRNAs with significant changes were retained for further analyses (Fig. 2). Five miRNA clusters were highlighted, and their target pathways were identified using IPA and represented in bar charts (Fig. 2). The first cluster (in purple) was characterized by miRNAs upregulated in young mice and downregulated in aged mice. Additionally, in young mice subjected to an HF diet, the expression of these miRNAs was mainly upregulated. The miRNAs in this cluster targeted several pathways linked to inflammation, senescence, or insulin signaling pathways (Fig. 2, cluster 1). The second cluster (illustrated by the green color) was characterized by an upregulation of miRNAs in aged mice with the CD diet compared with young mice and a tendency to decrease in the context of the HF diet. The miRNAs of this cluster targeted several pathways linked to inflammation, oxidative stress, diabetes, or retinoic acid signaling (Fig. 2, cluster 2). The third (in yellow) showed an upregulation of miRNAs in young HF-fed and aged CD-diet, and targeted pathways notably linked to apoptosis and inflammation (Fig. 2, cluster 3). Cluster 4 (in blue) was characterized by an upregulation of its miRNAs in obese mice, but no significant differences related to the age of mice. Additionally, miRNAs of this cluster targeted pathways dealing with NF-κB signaling and several nuclear receptors signaling pathways (Fig. 2, cluster 4). Finally, the fifth (represented by the red color) seems to follow the cytokines mRNA expression in WAT, with the upregulation of its miRNAs during obesity and aging, mainly with the combined effect of both. The miRNAs of this cluster targeted many inflammatory signaling pathways, diabetes-linked pathways, and cellular cycle regulation (Fig. 2, cluster 5).

**Figure 3:**
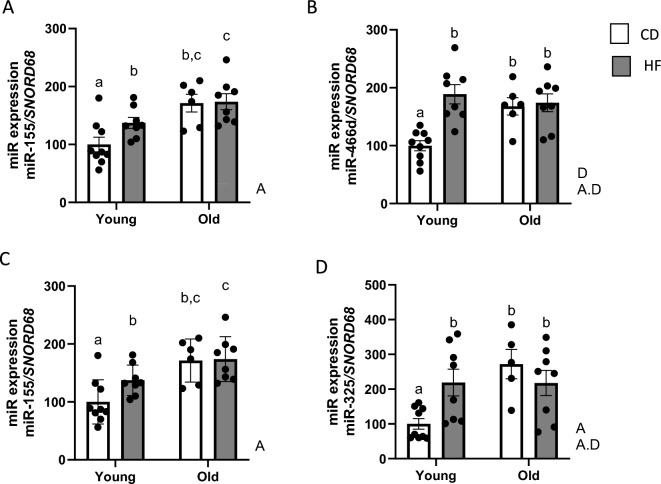
The combined effect of aging and obesity up-regulates inflammatory miRNAs in WAT. The graphic represents the miRNA expression of cluster 5 as (**A**) miR-155, (**B**) miR-466d, (**C**) miR-20b, and (**D**) miR-325 in WAT. Values are expressed as means ± SEM. Bars not sharing the same letter were significantly different in Fisher’s LSD post hoc test p < 0.05 between the control group (CD) and high-fat group (HF) for young and aged mice. A, age effect in two-way ANOVA analysis (p < 0.05); D, diet effect in two-way ANOVA analysis (p < 0.05); A⋅D, the interaction between time and diet in two-way ANOVA analysis (p < 0.05).

This cluster 5 is composed of miR-155, miR-466d, miR-20b, and miR-325 (Fig. 3). The mRNA expression levels of these miRNAs exhibited a significant increase in HF-fed young mice compared with young mice fed with a CD diet (Fig. 3A,B,D), except for miR-20b (Fig. 3C). Furthermore, these miRNAs were upregulated during aging, with no cumulative effect of aging and obesity, except for miR-155 (Fig. 3). Indeed, we observed a combined effect of aging and obesity, specifically on miR-155 expression, with an age effect also observed (Fig. 3A). We identified an interaction effect for all other miRNAs, and an age impact was observed for miR-325.

### The quantity of vitamin D was correlated to inflammation in WAT

**Figure 4:**
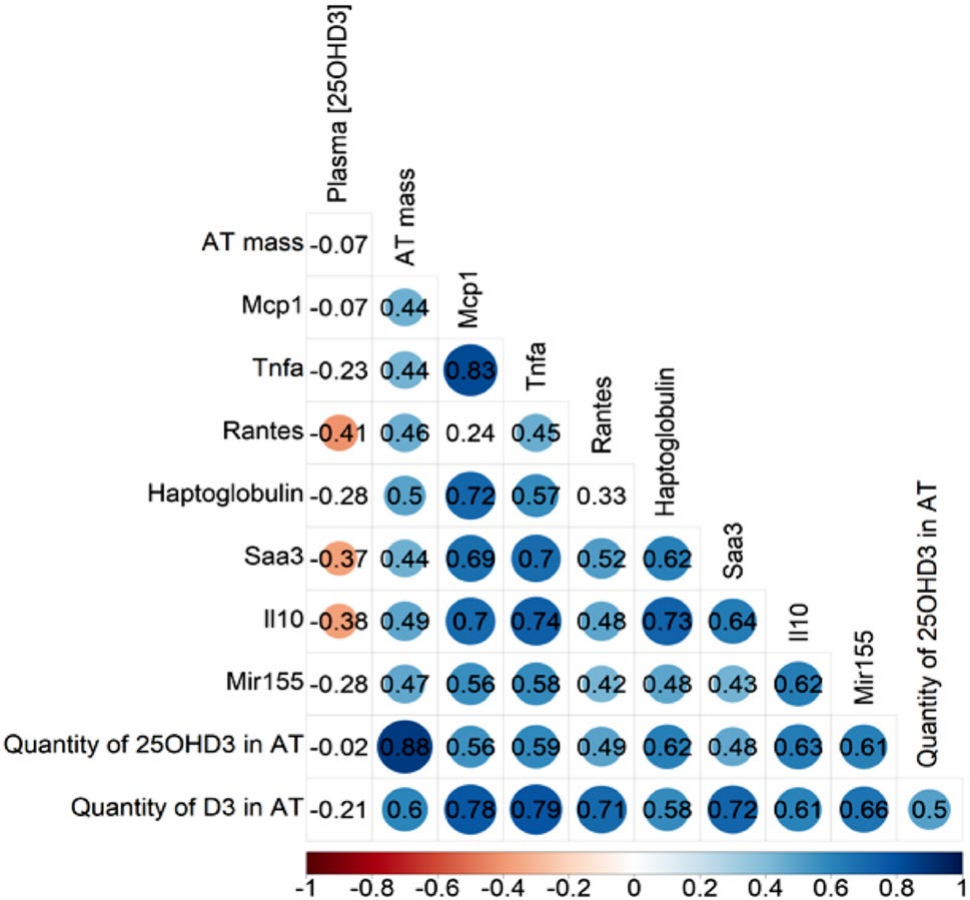
Correlation matrix between inflammation and vitamin D parameters. R software generated a correlation matrix; values are significant if the data are in a circle.

All the parameters previously measured in WAT were subjected to correlation analysis, as illustrated in Fig. 4. We observed an inverse correlation between the plasma 25(OH)D level and the mRNA expression of *Ccl5* (also known as *Rantes* in Fig. 4), *Saa3*, and *Il10* in WAT, with a trend (though not statistically significant) for the other inflammatory cytokines and miR-155 (Fig. 4). As anticipated, all inflammatory cytokines and miR-155 mRNA expression showed positive correlations. Additionally, the WAT mass (AT mass in Fig. 4) exhibited correlations with all the mRNA expression levels of these cytokines, miR-155, and the quantities of 25(OH)D and cholecalciferol in WAT. Of particular interest, we noticed a positive correlation between the quantity of 25(OH)D and all the inflammatory parameters within this correlation matrix (Fig. 4). Still, the 25(OH)D concentration in WAT was not correlated to inflammatory parameters (Supplemental data 4).

## Discussion

This study comprehensively examined adipose tissue inflammation under conditions of aging, obesity, and their combined impact. While systemic inflammation remained poorly impacted by aging or obesity in our conditions, adipose tissue inflammation was increased at both mRNA and miRNA levels according to a diet effect, an age effect, or a combined effect. Whereas plasma 25(OH)D levels were reduced by aging or obesity, cholecalciferol and 25(OH)D quantities in adipose tissue increased and were correlated with inflammatory markers.

In the present study, 2-month and 18-month-old mice were fed a control or HF diet for six months. Thus, at the end of the experimentation, young and aged mice were respectively 8 and 24 months old mice, which can be considered as a relevant model of aging since it is classically accepted that 18 months is the minimum age limit to simulate aging in humans with mice^34^. Additionally, it is crucial to notice that the two diets used in this study, respectively named AIN-93G for the control diet and 251HF for the HF diet, brought the same quantity of cholecalciferol (to avoid any confusing factor) as confirmed by measuring plasma cholecalciferol level of all groups. The HF diet containing 45% of energy from lipids induced obesity, as confirmed by the significant increase in body weight and peri-epididymal WAT of HF-fed mice. The impact of aging and obesity was also confirmed regarding 25(OH)D status. Indeed, during this experimentation, we observed that age, obesity, and the combined effect of both were associated with low levels of plasma 25(OH)D, as reported in the literature^27,28,35,36^. Moreover, the quantity of 25(OH)D in WAT, which was increased during obesity with a cumulative effect with aging, confirmed that aging and obesity are often linked with vitamin D metabolism disruptions, as previously reported^15,30,35^. Altogether, these observations validate our study model.

In addition to vitamin D metabolism disruption, aging and obesity are also frequently associated with systemic low-grade inflammation and adipose tissue inflammation^1,6,14,37^. Thus, we measured different inflammatory cytokines in the plasma and various mRNA coding for inflammatory markers in WAT. The quantities of inflammatory markers in the plasma were not significantly different between groups, suggesting a low impact of obesity and aging at the circulating level. Some studies have already reported that aging, in humans and mice, was associated with an increase in inflammatory cytokines^38–44^. Nevertheless, some studies have not found such results^45,46^, depicting that aging was not mandatory associated with increased systemic inflammation. Moreover, the same reports were observed during obesity^47–49^. The reasons for the discrepancy between studies are not currently understood. Still, they could be attributed to variations in experimental protocols, including the choice of mice genotype, duration of experimentations, age of mice, methodology of quantifications of cytokines, etc.) In addition, it is also essential to remember that we used an HF diet containing only 45% of energy from lipids, which corresponds to a diet leading to overweight or mild obesity. Thus, the lack of systemic inflammation could reflect the smooth obese phenotype implemented in the present study. The lack of inflammation in old mice is more surprising since our 24-month-old mice must be considered an excellent aging model, and the cytokines we measured are classically deregulated in aged mice^38^. The origin of such discrepancies should be investigated.

Adipose tissue is well known to display an increased inflammatory status during aging or obesity in mice^2,3,18,19,24,37^. In agreement, we confirmed in our model that mRNA expressions of pro-inflammatory cytokines were induced. Moreover, a solid combined impact of aging and obesity for *Tnf* and *Ccl5* mRNA expression was observed with a trend for Mcp1. It is important to note that obesity during aging has been linked to many aging-associated diseases, which could explain this cumulative effect. Moreover, it is known that during the aging process, there is a transition in fat distribution from subcutaneous adipose tissue to visceral fat depots^10^, which could contribute to this combined effect. Reciprocally, obesity is also linked to inflammation of fatty tissue, notably represented by the induction of *Tnf* mRNA expression, which could accelerate the aging of adipose tissue and increase the production of pro-inflammatory cytokines during aging^6,50,51^. Nevertheless, it is essential to mention as a limitation that only mRNA has been quantified, and even if it has been previously highlighted that mRNA and protein levels were correlated in adipose tissue^24^, it would be interesting to confirm these data at the protein level. In addition, the lack of correlation with plasma inflammatory markers is also surprising but still of interest for local adipose tissue biology.

Several studies have shown inflammation during obesity or aging-induced miRNA expression, notably in WAT and adipocytes^6,8,9,18,20^. Thus, we assessed the expression of an inflammatory miRNA panel in WAT and generated a heatmap with the miRNAs exhibiting statistically significant changes. Five distinct inflammatory miRNA clusters were identified, each exhibiting specific regulation patterns and targeting different signaling pathways. The first cluster contained miRNAs down-regulated during aging. Interestingly, we noticed the one related to calcium transport and signaling among affected pathways. In agreement, it has already been described that the calcium pathways were disrupted in older people, notably due to an increase in parathormone in aging^52^, an indirect down-regulator of 25-hydroxylation of vitamin D^53^. Thus, this impact of parathormone through the calcium pathway could contribute to our models' observed 25(OH)D circulating levels. Moreover, we identified senescence and inflammation among affected pathways, consistent with mice's aging process and adipose tissue inflammation as previously described above. Finally, this cluster also targeted insulin pathways, which are closely associated with the development of diabetes in aging individuals, as reported in the literature^54^. An up-regulation of miRNAs in aged CD-fed mice and a modest HF diet effect on miRNA expression mainly characterized the second cluster. In agreement, the S100 family signaling pathway targeted by the differentially regulated miRNAs of this cluster is known to activate inflammatory NF-κB pathways. Interestingly, it has been described that miR-146a, which belongs to the cluster (expression in supplemental data 2), is strongly related to NF-κB pathways during aging^55^ and could thus participate in the inflammation and oxidative pathways (also targeted by this cluster) in our model. Finally, this cluster seems to be linked to diabetes pathways. Such an assumption is in line with our results (data not shown) and in agreement with the observed higher prevalence of diabetes during aging and obesity^54,56,57^.

Cluster 4 showed an up-regulation of miRNAs in obese mice (both young and aged mice), illustrating a diet effect. These miRNAs, including miR-221 and miR-222, targeted the NF-κB signaling, which is a crucial mediator of obesity-related inflammation^58–60^. Moreover, among affected pathways, several nuclear receptors signaling were impacted, notably the vitamin D receptor (VDR/RXR) signaling, which could participate in the increased adipose tissue inflammation^14,18,20,24^. The fifth cluster appeared to follow the mRNA expression patterns of cytokines in WAT, with miRNAs being upregulated during obesity and aging, mainly in the combined effect of both. Among the pathways targeted, the diabetes-linked pathways were impacted. Moreover, several inflammatory signaling pathways involved in the inflammatory process were also targeted. This result could be explained by the presence of miR-155 in this cluster, which was involved in inflammatory pathways^6,18^. Indeed, studies have demonstrated an upregulation of miR-155 in response to inflammatory stimulations during aging and obesity^20,61^. Such induction of miR-155 in our experimental conditions could vigorously participate in adipose tissue inflammation. Interestingly, miR-155 was the only one with the combined effect of aging and obesity. The origin of this combined effect could be explained by our team's recent study, which showed that miR-155 was notably induced by TNF^6^. As reported in the correlation matrix, we observed a strong correlation between miR-155 and *Tnf*. In addition, miR-155 was correlated with all the mRNA expression of inflammatory cytokines, and these inflammatory parameters were also associated with the mass of WAT and the quantity of cholecalciferol and 25(OH)D3 but not with the concentration of 25(OH)D in the WAT (Supplemental data 4). The fact that miR-155 was correlated with WAT mass agreed with the induced inflammatory process associated with fat mass accretion. It is more surprising that this proinflammatory miR-155 was also related to the quantity of 25(OH)D, which is considered to display proinflammatory effects^12,13,18,19^. Nevertheless, the 25(OH)D quantity was also correlated to fat mass. Thus, the relationship between miR-155 expression and 25(OH)D quantity was probably driven only by increased fat mass. Nevertheless, the accumulation of 25(OH)D and cholecalciferol in WAT, even if not sufficient to blunt WAT inflammation during obesity and aging, may represent a mechanism that limits the extent of the inflammatory process. Without 25(OH)D and cholecalciferol stored in WAT, we could expect inflammation in WAT to be even higher.

The present study has some limitations. Indeed, it would be interesting to evaluate all these parameters in female mice to highlight the impact of gender on the inflammation of fatty tissue, as it has been reported that vitamin D status differs with gender^62,63^. In addition, in adipose tissue, only mRNA and miRNA were quantified, and it would be of interest to quantify several proteins of inflammation in adipose tissue. In the plasma, we did not observe clear inflammation in both obesity and aging. This point is surprising but could be due to the type of diet we used to induce obesity (containing only 45% of energy from lipids). Nevertheless, the lack of inflammation in aged mice is presently not explained.

In conclusion, our study highlighted the inflammatory state in WAT in our aging model alone or combined with obesity, as evidenced by increased mRNA expression of pro-inflammatory cytokines and miRNAs. The combination of aging and obesity significantly impacted *Tnf* mRNA levels and miR-155 expression, which could target the NF-κB pathways. Additionally, we observed a correlation between inflammatory markers and the quantity of cholecalciferol and 25(OH)D present in WAT. This association suggests a potential protective mechanism of vitamin D to limit inflammation during adipose accretion, which aligns with previous research conducted by our team^18,19,24^. These findings shed light on the intricate interplay between aging, obesity, inflammation, and vitamin D metabolism in WAT, offering valuable insights into potential therapeutic avenues for managing these health challenges.

### Supplementary Information

Supplementary Information.

## Data Availability

The data supporting this study's findings are available from the corresponding author upon reasonable request.
