# Supplementary material for: Aging alone or combined with obesity increases white adipose tissue inflammatory status in male mice
Source: Sci Rep. 2024 Jul 15;14:16268. doi: 10.1038/s41598-024-67179-3 (PMC11251036; doi:10.1038/s41598-024-67179-3)
Supplement: Supplementary file 1 — Supplementary Information. [file 41598_2024_67179_MOESM1_ESM.docx]

***Supplemental data 1. Effect of aging and diet on body weight gain, fat mass and adiposity index.*** *(A) Final body weight gain with delta. Values are expressed as means ± SEM. Bars not sharing the same letter were significantly different in Tukey post hoc test p < 0.05 between control group (CD) and high fat group (HF) for young and aged mice. A, age effect in two-way ANOVA analysis (p < 0.05); D, diet effect in two-way ANOVA analysis (p < 0.05); A.D, interaction between time and diet in two-way ANOVA analysis (p < 0.05).*

**

***Supplemental data 2. Heatmap of miRNA in WAT was generated by MetaboAnalyst software.***


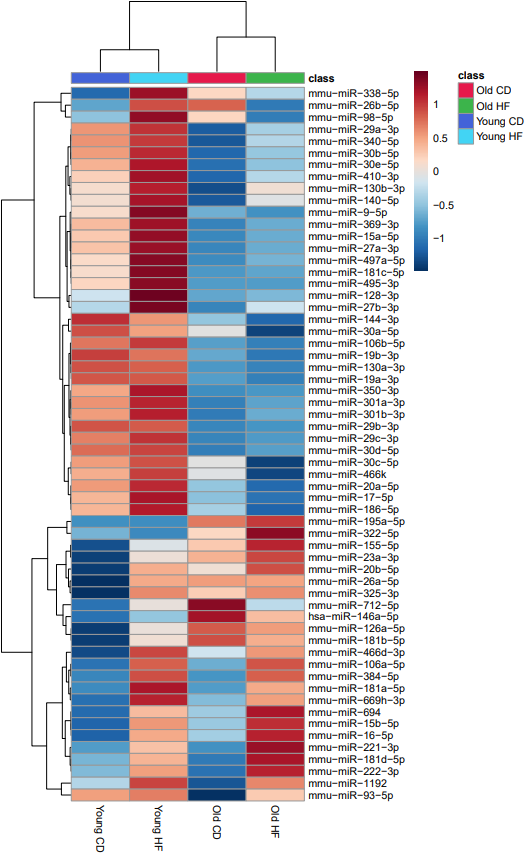


*****Supplemental data 3. Table of inflammatory miRNA expression in WAT.*** *Values are expressed as means ± SEM. Values not sharing the same letter were significantly different in Fisher’s LSD post hoc test p < 0.05 between control group (CD) and high fat group (HF) for young and aged mice. A, age effect in two-way ANOVA analysis (p < 0.05); D, diet effect in two-way ANOVA analysis (p < 0.05); A.D, interaction between time and diet in two-way ANOVA analysis (p < 0.05).*

***Supplemental data 4. Pearson matrix correlation between concentration of 25(OH)D (ng/ml) and inflammatory parameters in WAT.***

***
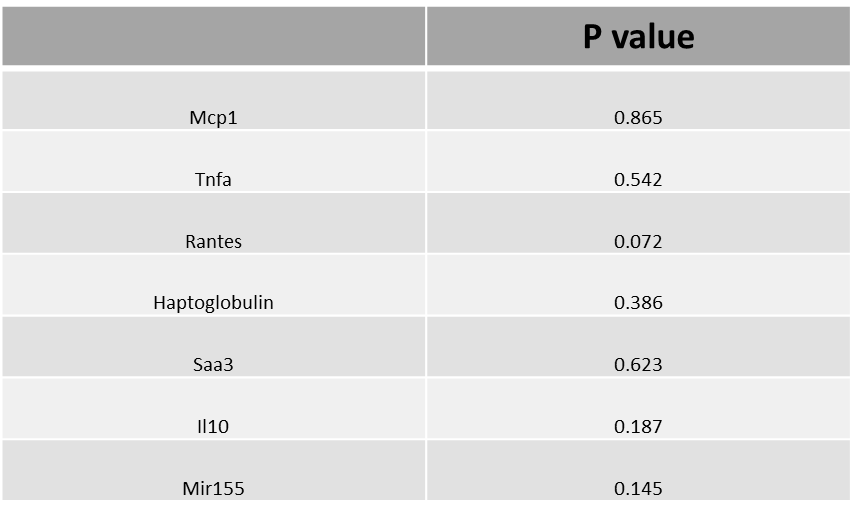
***
